# Mineralocorticoid and glucocorticoid receptors differentially regulate NF-kappaB activity and pro-inflammatory cytokine production in murine BV-2 microglial cells

**DOI:** 10.1186/1742-2094-9-260

**Published:** 2012-11-28

**Authors:** Boonrat Chantong, Denise V Kratschmar, Lyubomir G Nashev, Zoltan Balazs, Alex Odermatt

**Affiliations:** 1 Division of Molecular and Systems Toxicology, Department of Pharmaceutical Sciences, University of Basel, Klingelbergstrasse 50, CH-4056, Basel, Switzerland

**Keywords:** Mineralocorticoid receptor, Glucocorticoid receptor, 11β-hydroxysteroid dehydrogenase, Inflammation, Interleukin-6, NF-κB

## Abstract

**Background:**

Microglia, the resident macrophage-like cells in the brain, regulate innate immune responses in the CNS to protect neurons. However, excessive activation of microglia contributes to neurodegenerative diseases. Corticosteroids are potent modulators of inflammation and mediate their effects by binding to mineralocorticoid receptors (MR) and glucocorticoid receptors (GR). Here, the coordinated activities of GR and MR on the modulation of the nuclear factor-κB (NF-κB) pathway in murine BV-2 microglial cells were studied.

**Methods:**

BV-2 cells were treated with different corticosteroids in the presence or absence of MR and GR antagonists. The impact of the glucocorticoid-activating enzyme 11β-hydroxysteroid dehydrogenase type 1 (11β-HSD1) was determined by incubating cells with 11-dehydrocorticosterone, with or without selective inhibitors. Expression of interleukin-6 (IL-6), tumor necrosis factor receptor 2 (TNFR2), and 11β-HSD1 mRNA was analyzed by RT-PCR and IL-6 protein expression by ELISA. NF-κB activation and translocation upon treatment with various corticosteroids were visualized by western blotting, immunofluorescence microscopy, and translocation assays.

**Results:**

GR and MR differentially regulate NF-κB activation and neuroinflammatory parameters in BV-2 cells. By converting inactive 11-dehydrocorticosterone to active corticosterone, 11β-HSD1 essentially modulates the coordinated action of GR and MR. Biphasic effects were observed for 11-dehydrocorticosterone and corticosterone, with an MR-dependent potentiation of IL-6 and tumor necrosis factor-α (TNF-α) expression and NF-κB activation at low/moderate concentrations and a GR-dependent suppression at high concentrations. The respective effects were confirmed using the MR ligand aldosterone and the antagonist spironolactone as well as the GR ligand dexamethasone and the antagonist RU-486. NF-κB activation could be blocked by spironolactone and the inhibitor of NF-κB translocation Cay-10512. Moreover, an increased expression of TNFR2 was observed upon treatment with 11-dehydrocorticosterone and aldosterone, which was reversed by 11β-HSD1 inhibitors and/or spironolactone and Cay-10512.

**Conclusions:**

A tightly coordinated GR and MR activity regulates the NF-κB pathway and the control of inflammatory mediators in microglia cells. The balance of GR and MR activity is locally modulated by the action of 11β-HSD1, which is upregulated by pro-inflammatory mediators and may represent an important feedback mechanism involved in resolution of inflammation.

## Background

Glucocorticoids are essential for the coordinated regulation of metabolic and immune responses. They are well known due to their potent anti-inflammatory and immune suppressive effects, and therefore widely used in clinics to treat inflammatory and autoimmune diseases. Increasing evidence indicates that endogenously occurring glucocorticoids can, in some situations, stimulate inflammation by enhancing the production of pro-inflammatory mediators and promoting oxidative stress [1-3]. The underlying mechanisms and the role of corticosteroid receptors are, however, not fully understood.

Glucocorticoids exert their effects mainly by activating glucocorticoid receptors (GR) and mineralocorticoid receptors (MR). A comparison of corticosterone binding to MR and GR in rat hippocampal preparations revealed a 10-fold higher affinity for MR (K_d_ = 0.5 nM) compared with GR (K_d_ = 5.0 nM) [4,5]. Importantly, hippocampal MR was occupied about 80% while GR was occupied 10% only if samples were taken at the nadir of the hypothalamus-pituitary adrenal (HPA) axis diurnal rhythm in the morning. GR binding increased at higher corticosterone levels, suggesting that GR is activated during stress [6,7] and potentially also at diurnal peak levels. Differential binding by MR and GR in various brain cells needs to be considered in order to understand effects of glucocorticoids in the CNS [8-10].

In classical mineralocorticoid target tissues involved in electrolyte and volume regulation, such as renal cortical collecting ducts, distal colon, and salivary and sweat glands, MR is co-expressed with 11β-hydroxysteroid dehydrogenase 2 (11β-HSD2), which converts active (corticosterone, cortisol) into inactive glucocorticoids (11-dehydrocorticosterone, cortisone) [11-13]. The close proximity of 11β-HSD2 and MR has been proposed to prevent MR activation by glucocorticoids, thereby rendering specificity of the receptor to aldosterone [14]. Deficient 11β-HSD2 activity has been shown to result in sodium and water retention, causing edema formation and hypertension [15-17]. To avoid these adverse effects caused by excessive activation of renal and intestinal MR, synthetic glucocorticoids selectively activating GR have been designed and are widely used in therapy of inflammatory and autoimmune diseases [18,19].

Besides, MR has important functions in vascular cells, adipocytes, osteocytes, neutrophils, dendritic cells, and macrophage [11]. In the brain, MR plays an important role in hippocampal neurons and in immune cells [20-22]. Glucocorticoids are essentially involved in the modulation of the coordinated action of monocytes, macrophages, astrocytes, and microglia cells during inflammation in the brain [23]. Interestingly, in macrophage and microglia cells, MR and GR are co-expressed in the presence of 11β-HSD1, which converts inactive 11-dehydrocorticosterone and cortisone into active corticosterone and cortisol [11]. Since circulating glucocorticoid concentrations are 100 to 1,000 times higher than those of aldosterone, MR activity is most likely controlled by glucocorticoids but not aldosterone in these cells under physiological conditions [24].

So far, only few cell lines have been reported that express MR, GR, and 11β-HSD1 at sufficient levels, allowing to investigate the functional interactions between the two corticosteroid receptors and to assess the role of 11β-HSD1 in regulating their activities. In the present study, we show that the murine microglial cell line BV-2, which is considered to be a suitable model of microglia to study inflammatory parameters [25], expresses MR, GR, and 11β-HSD1. We compared the effects of the endogenous glucocorticoids 11-dehydrocorticosterone (which requires activation by 11β-HSD1) and corticosterone, the mineralocorticoid aldosterone and the synthetic glucocorticoid dexamethasone on NF-κB activation and IL-6 expression in the presence or absence of MR and GR antagonists. Moreover, the impact of pro-inflammatory cytokines and corticosteroids on the expression of TNFR2 and 11β-HSD1 was investigated.

## Methods

### Materials

Fetal bovine serum (FBS) was purchased from Atlanta Biologicals (Lawenceville, GA, USA) and other cell culture media and supplements from Invitrogen (Carlsbad, CA, USA). *Escherichia coli* 0111:B4 lipopolysaccharide (LPS), TNFα, and IL-6 were purchased from Sigma-Aldrich (St. Louis, MO, USA), Cay-10512 was from Cayman Chemicals (Hamburg, Germany), [1,2-^3^H]-cortisone from American Radiolabeled Chemicals (St. Louis, MO, USA), IL-6 ELISA kit from BD Biosciences (Allschwil, Switzerland), and the HCS kit for evaluation of NF-κB activation (K010011) was obtained from Cellomics ThermoScientific (Pittsburgh, PA, USA). Antibodies against HDAC-1, TNFR2, NF-κB subunit p65, and phosphorylated p65 were obtained from Cell Signaling Technology (Danvers, MA, USA). Antibody against β-actin and goat anti-rabbit IgG-HRP were obtained from Santa Cruz Biotechnology (Santa Cruz, CA, USA).

### Cell culture

The immortalized mouse microglial cell line BV-2, developed by Blasi *et al*. [26,27], was kindly provided by Professor Wolfgang Sattler, University of Graz, Graz, Austria. Cells were cultivated in RPMI-1640 medium supplemented with 10% FBS, 2 mM glutamine, 100 μg/mL streptomycin, 100 U/mL penicillin, 0.1 mM non-essential amino acids, and 10 mM HEPES, pH 7.4. Prior to treatment, cells were seeded in 96-well (1 × 10^3^ cells), 24-well (2 × 10^4^ cells), 12-well (5 × 10^5^ cells), or 6-well (1 × 10^6^ cells) plates in Dulbecco’s modified Eagle’s medium (DMEM) supplemented as indicated above and incubated for 24 h at 37°C. All experiments were performed under the permission A070126 by the Swiss Federal Department of Environment BAFU.

### Quantification of mRNA expression

Total RNA from treated cells was isolated using Trizol reagent according to the protocol from the manufacturer (Invitrogen). The concentration and purity of the total RNA was assessed by a NanoDrop® spectrophotometer. A 260/280 nm absorbance ratio of 1.8 or higher was accepted for purity of total RNA. Total RNA was used for cDNA synthesis with oligo-dT primers and the Superscript-III first-strand synthesis system (Invitrogen). Total RNA (0.5 μg) and oligo-dT primers (0.5 μg) were incubated for 5 min at 56°C, followed by addition of 4.90 μL of 5 × first strand buffer, 1.5 μL of 0.1 M dithiothreitol (DTT), 0.5 μL of RNAse H inhibitor (50 U), 1 μL of 10 mM dNTPs, and 0.1 μL of Supersript® Reverse Transcriptase III (total reaction volume 24.5 μL). The reaction was performed for 1 h at 42°C, followed by adding 80 μL of DEPC-treated water and storage at −20°C until further use. Relative quantification of mRNA expression levels was performed by real-time RT-PCR using the KAPA SYBR® FAST qPCR Kit (Kapa Biosystems, Boston, MA, USA) and gene-specific oligonucleotide primers. Quantitative real-time RT-PCR was performed in a final volume of 20 μL and using a Rotor-Gene 6000 thermal cycler (Corbett Research, Sydney, Australia). The following amplification procedure was applied: initial denaturation for 15 min at 95°C, followed by 40 cycles of denaturation for 15 s at 94°C, annealing for 30 s at 56°C, and extension for 30 s at 72°C. Three replicates were analyzed per sample. Relative gene expression compared with the internal control GAPDH was determined using the 2^-ΔΔCT^ method [28]. The oligonucleotide primers for real-time RT-PCR are listed in Additional file 1: Table S1.

### Determination of IL-6 protein expression

Cells grown in 96-well plates were treated for 24 h with the compounds indicated, and cell-free supernatants were collected. IL-6 protein was measured using an ELISA kit from BD Biosciences. Briefly, a 96-well plate was coated with capture antibody overnight at 4°C, followed by washing five times with PBS-T (PBS containing 0.05% Tween-20). The wells were blocked with assay diluents for 1 h and washed five times. Standards and collected culture supernatants were added to the appropriate wells and samples were incubated for 2 h. After rinsing five times with PBS-T, each well was incubated with detection antibody for 1 h. After washing, avidin-HRP was added for 30 min. After rinsing seven times, each well was incubated with substrate solution for 30 min in the dark. Reaction was stopped by adding 1 M H_3_PO_4_, and the plate was analyzed by measuring absorbance at 450 nm and subtracting the values at 570 nm using a UV-max kinetic microplate reader (Molecular Device, Devon, UK). IL-6 protein concentrations were calculated according to the standard curve of purified mouse IL-6.

### Determination of NF-κB localization by fluorescence microscopy

BV-2 cells were grown on poly-L-lysine coated glass slides in 6-well plates. Following treatment, the medium was removed and cells were fixed and stained using Cellomics®NF-κB p65 activation HCS kit (Cellomics ThermoScientific, Pittsburgh, PA, USA) according to the manufacturer’s instructions. Briefly, cells were fixed with 4% paraformaldehyde in PBS for 15 min at 25°C, washed three times with PBS, and permeabilized with PBS containing 0.5% Triton X-100 for 10 min. After three washes with PBS and blocking with 1% fatty acid-free bovine serum albumin for 1 h the samples were incubated with rabbit polyclonal antibody against the p65 subunit of NF-κB (1:500) for 2 h at 37°C. After washing, slides were incubated in the dark with blocking buffer containing goat anti-rabbit IgG conjugated to Alexa Fluor 488 and Hoechst 33342 dye for 1 h. Slides were washed with PBS and mounted with Mowiol® 4–88 antifade reagent (Hoechst, Frankfurt, Germany) on glass slides. Cells were analyzed using a confocal laser scanning microscope Olympus Fluoview FV1000. Images were captured using a magnification of 400×. The excitation and emission wavelengths were 488 nm and 510 nm, respectively.

Alternatively, cells were grown in 96-well plates, fixed and stained using Cellomics NF-κB p65 activation HCS reagent kit. The cells were soaked in PBS at 4°C until imaging procedure. The assay plate was analyzed on a Cellomics ArrayScan HCS reader. The Cytoplasm to Nucleus Translocation BioApplication software was used to calculate the ratio of cytoplasmic and nuclear NF-κB intensity [29]. The average intensity of 500 objects (cells) per well was quantified (magnification 200×). Nuclear stain (Hoechst 33342) was in channel 1 and NF-κB was visualized in channel 2. In channel 2 an algorithm was utilized to identify the nucleus and surrounding cytoplasm in each cell; this analysis method reports the average intensity within each nuclear mask as well as the total of the nuclear and cytoplasmic intensity. The results were reported as percentage of nuclear intensity from total intensity. All treatments were performed in triplicate.

### Preparation of whole cell lysates and cytoplasmic and nuclear fractions

For preparation of whole cell lysates, cells grown in 6-well plates were washed twice with ice-cold PBS and lysed in cold RIPA buffer (Sigma-Aldrich) supplemented with protease inhibitor cocktail (Roche Diagnostics, Rotkreuz, Switzerland). The extract was centrifuged at 10,000 × g for 15 min at 4°C to remove cell debris.

For preparation of cytoplasmic and nuclear fractions cells were washed twice with PBS, harvested in 500 μL PBS, followed by centrifugation at 450 × g for 5 min. Cell pellets were washed once with PBS, transferred to 1.5-mL tubes and pelleted again at 1,000 × g for 5 min. The cells were lysed by gentle resuspension in 200 μL lysis buffer containing 0.01 M DTT and protease inhibitors, followed by incubation on ice for 15 min. IGEPAL CA-630 solution (Sigma-Aldrich) was added at 0.6% v/v final concentration and samples were vigorously mixed for 10 s, followed by centrifuging at 10,000 × g for 30 s. Supernatants (cytoplasmic fractions) were transferred to new tubes. The nuclear pellets were washed once with 100 μL PBS, pelleted at 450 × g for 5 min, and resuspended in 100 μL of extraction buffer containing 0.01 M DTT and protease inhibitors. Nuclear suspension was agitated for 30 min and centrifuged at 12,000 × g for 5 min. Nuclear fractions were frozen at −80°C and thawed on ice to increase extraction of nuclear proteins from the insoluble material. Nuclear samples were then sonicated on ice three times for 5 s each to obtain the final nuclear fractions.

### Detection of protein expression

Protein concentrations were determined using the Pierce® BCA protein assay kit (Thermo Scientific) according to the manufacturer’s instructions and bovine serum albumin as standard. Cytoplasmic, nuclear, or whole cell extracts (30 μg per sample) were resolved on 8% sodium dodecyl sulfate-polyacrylamide gel electrophoresis (SDS-PAGE). Proteins were then transferred to 0.2 μm polyvinylidene difluoride (PVDF) membranes (Bio-Rad Laboratories, Hercules, CA, USA). Membranes were incubated in Tris-buffered saline, pH 7.4, containing 0.1% Tween-20 (TBS-T), and supplemented with 5% non-fat milk overnight at 4°C for blocking. The blots were then incubated with primary antibodies against the p65 subunit of NF-κB (1:500), the phosphorylated form of p65 (1:500), TNFR2 (1:1,000), HDAC1 (1:1,000) or β-actin (1:1,000) for 2 h at room temperature. After washing with TBS-T, blots were incubated with goat anti-rabbit IgG-horseradish peroxidase or anti-mouse IgG-horseradish peroxidase (1:5,000) for 1 h at room temperature. Blots were washed with TBS-T and immunolabeling was visualized using enhanced chemiluminescence HRP substrate (Millipore, Billerica, MA, USA) according to the manufacturer’s instructions using a Fujifilm LAS-4000 detection system (Bucher Biotec, Basel, Switzerland). β-actin was used as loading control for cytoplasmic and whole cell protein extracts. HDAC1 was used as loading control for nuclear fractions.

### 11β-HSD1 activity measurements in intact BV-2 cells

BV-2 cells were grown in 24-well plates and treated with 20 ng/mL LPS in the presence or absence of 200 nM cortisone for 24 h. Medium was removed and replaced by assay medium containing 50 nM cortisone with 10 nCi [^3^H] cortisone as tracer. Following incubation for 12 h at 37°C, medium was collected and steroids were extracted with ethyl acetate. The extracts were dried under vacuum, reconstituted with methanol containing a mixture of 2 mM unlabeled cortisone and cortisol, and steroids were separated in a chloroform/methanol solvent system (9:1, v/v). The conversion of cortisone to cortisol was determined by scintillation counting.

### Statistical analysis

The data were analyzed using one-way ANOVA (V5.00; GraphPad Prism Software Inc., San Diego, CA, USA). When ANOVA showed significant differences between groups, Tukey’s post hoc test was used to determine whether specific pairs of groups showed statistically significant differences. *P* < 0.05 was considered statistically significant.

## Results

### Potentiation of LPS-induced pro-inflammatory cytokine expression by low concentrations of endogenous glucocorticoids

To test whether the murine microglia cell line BV-2 might represent a suitable cell system to study the role of corticosteroid receptors and local glucocorticoid activation on pro-inflammatory cytokine production, we first determined the mRNA expression of GR, MR, and 11β-HSD1 using real-time RT-PCR. Substantial levels of mRNA expression for all three genes were detected. Approximately 10-fold higher GR mRNA than MR mRNA expression was obtained, in line with evidence for an order of magnitude higher expression of GR compared with MR in the kidney [30]. Thus, the expression pattern suggests that BV-2 cells can be used to investigate mechanisms of corticosteroid-mediated modulation of NF-κB activation and pro-inflammatory cytokine expression.

**Figure 1:**
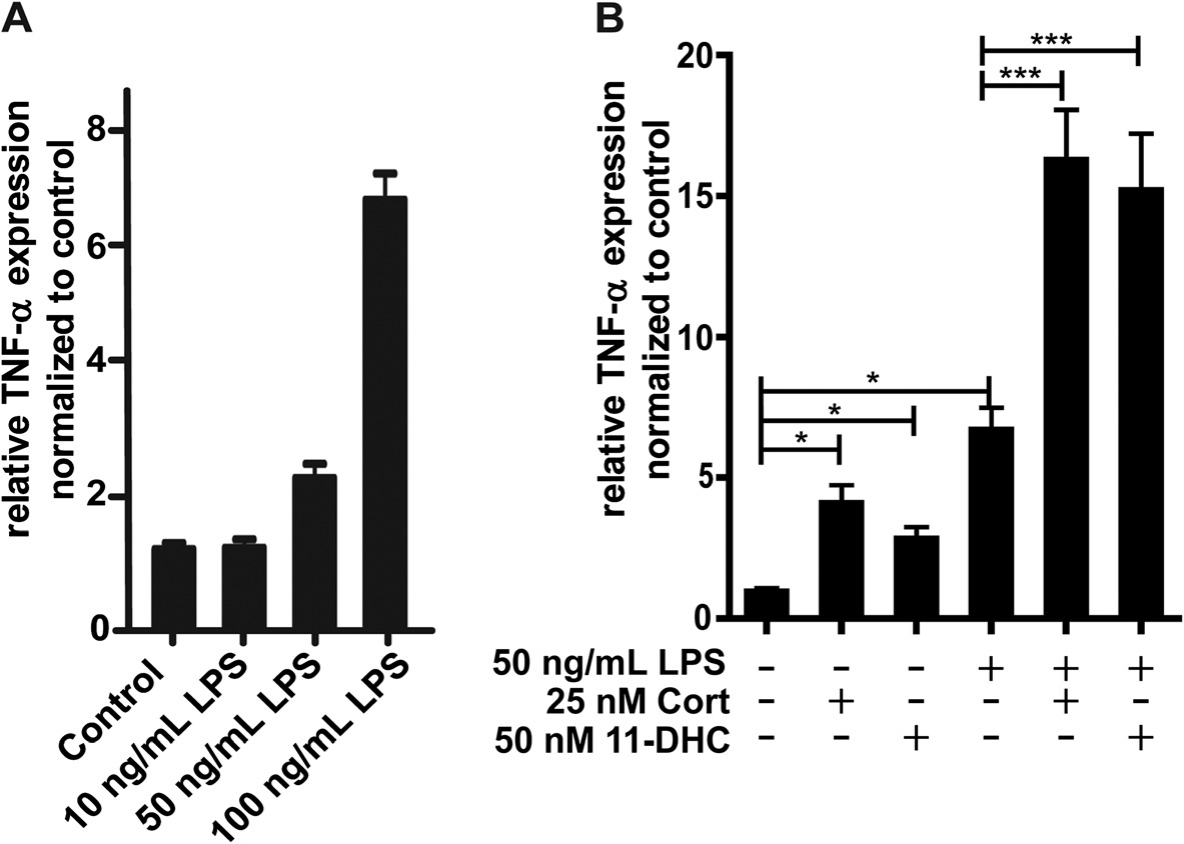
**Potentiation of LPS**-**induced TNF**-**α mRNA expression by low concentrations of corticosterone.** (**A**) BV-2 cells were incubated with 10, 50, and 100 ng/mL LPS for 24 h. (**B**) Cells were exposed to 50 nM 11-dehydrocorticosterone, which requires conversion to corticosterone by 11β-HSD1, or 25 nM corticosterone for 24 h prior to incubation with 50 ng/mL LPS for another 24 h. TNF-α mRNA expression was measured by real-time RT-PCR. Data (mean ± SD of three independent experiments) represent ratios of TNF-α mRNA to GAPDH control mRNA from treated cells normalized to the values obtained from cells incubated with vehicle (DMSO). *P <0.05, ***P <0.005.

BV-2 cells are sensitive to LPS, and incubation with LPS resulted in a concentration-dependent increase in the expression of TNF-α mRNA (Figure 1A). It is important to note that the absolute response depends on the batch of LPS and on batch and state of BV-2 cells. The three independent experiments shown in Figure 1A were performed with different batches of LPS and BV-2 cells than the three independent experiments shown in Figure 1B. Another factor influencing the absolute response values is the composition of the culture medium. Performing the experiments shown in Figure 1B in medium with charcoal-treated FBS resulted in almost two-fold higher TNF-α mRNA expression levels, whereby the relative expression changes were comparable (data not shown). As shown in Figure 1B preincubation of cells for 24 h with 25 nM corticosterone, corresponding to low physiological glucocorticoid concentrations, resulted in a pronounced stimulation of TNF-α expression upon treatment with LPS. Comparable effects, although somewhat less efficient, were observed when using 11-dehydrocorticosterone as substrate. This suggests a pro-inflammatory effect of low endogenous glucocorticoid concentrations and a role of the glucocorticoid-activating enzyme 11β-HSD1 in stimulating inflammation.

**Figure 2:**
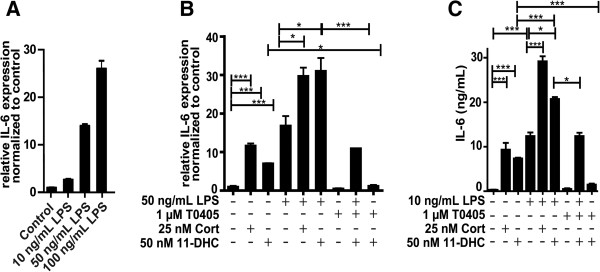
**Inhibition of 11β**-**HSD1 reversed the 11**-**dehydrocorticosterone**-**dependent stimulation of LPS induced IL**-**6 expression.** (**A**, **B**) IL-6 mRNA expression was measured by real-time RT-PCR. Ratios of IL-6 mRNA to GAPDH control mRNA of treated cells were normalized to values obtained from cells incubated with vehicle (DMSO). (A) BV-2 cells were treated for 24 h with LPS. (B) Cells were pretreated for 24 h with 25 nM corticosterone or with 50 nM 11-dehydrocorticosterone in the presence or absence of 1 μM of the 11β-HSD1 inhibitor T0504, followed by incubation with 50 ng/mL LPS for an additional 24 h. (**C**) IL-6 protein levels were quantified by ELISA. Cells were treated for 24 h with 25 nM corticosterone or with 50 nM 11-dehydrocorticosterone in the presence or absence of 1 μM T0504, followed by 10 ng/mL LPS for an additional 24 h. Data normalized to control represent mean ± SD from three independent experiments. *P <0.05, ***P <0.005.

We measured IL-6 expression as an accepted read-out to assess the sensitivity of exposure to LPS and subsequent activation of NF-κB [31]. LPS induced IL-6 expression in a concentration-dependent manner (Figure 2A). Preincubation with 25 nM corticosterone potentiated IL-6 expression (Figure 2B). A similar stimulation of IL-6 expression was observed upon preincubation with 50 nM 11-dehydrocorticosterone, an effect which was reversed by the two structurally distinct selective 11β-HSD1 inhibitors BNW-16 [32] (data not shown) and T0504 (Figure 2B, also known as Merck-544 [33,34]). The impact of LPS and glucocorticoids on IL-6 protein expression was assessed by ELISA. Increased IL-6 protein was observed upon treatment of cells with LPS, whereby the effect on protein expression was more pronounced than the effect on mRNA levels, suggesting enhanced translation and/or protein stability in addition to enhanced gene expression. Importantly, both preincubation with corticosterone and 11-dehydrocorticosterone further increased IL-6 expression, whereby the effect of the latter was blocked by 11β-HSD1 inhibitors (Figure 2C). In the absence of LPS, incubation of BV-2 cells with 25 nM corticosterone and 50 nM 11-dehydrocorticosterone both enhanced IL-6 expression. As expected, the effect of 11-dehydrocorticosterone was abolished by 11β-HSD1 inhibition.

### MR and GR differentially modulate the IL-6 expression

**Figure 3:**
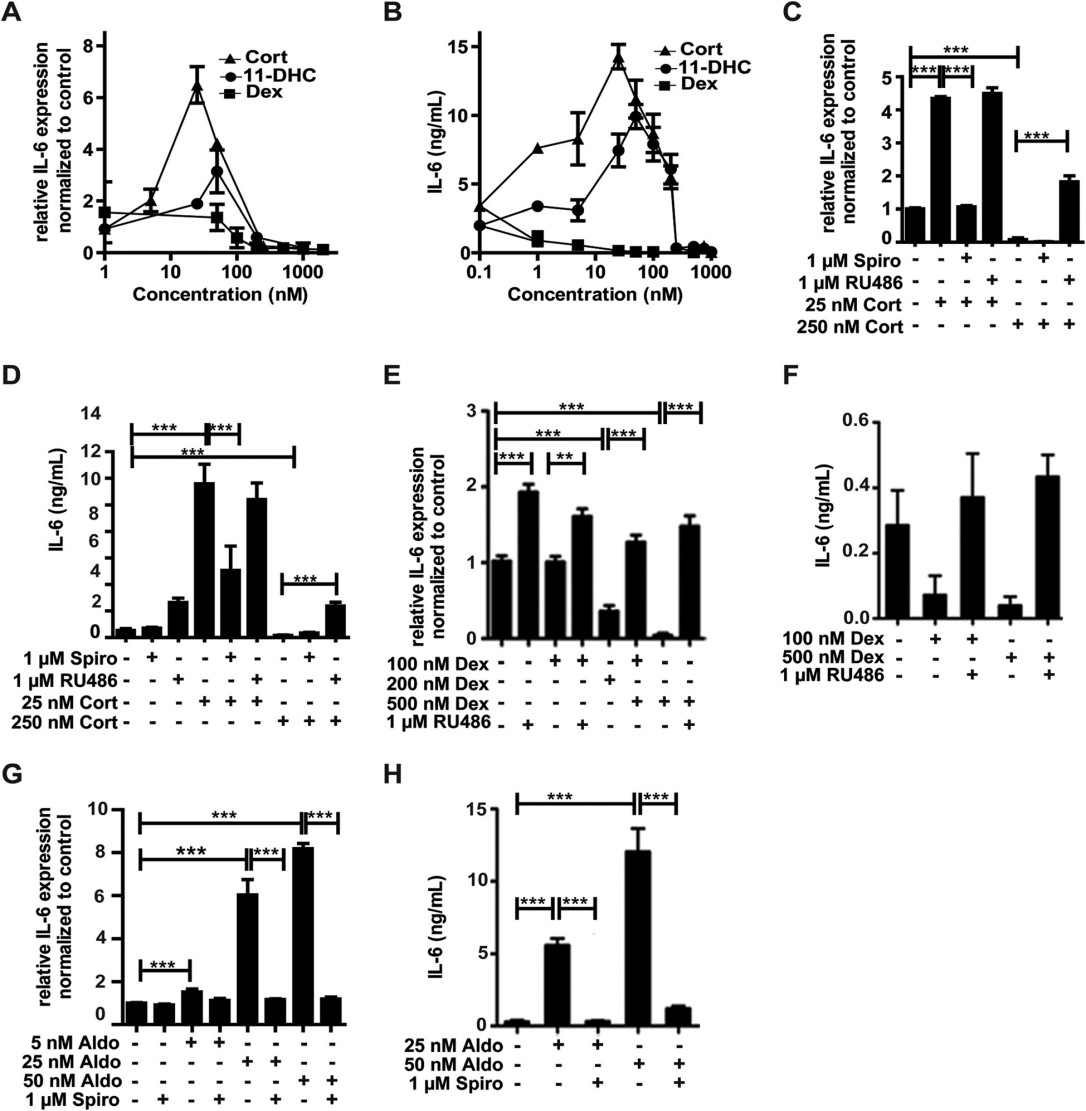
**Differential modulation of IL**-**6 expression by MR and GR.** BV-2 cells were treated with various concentrations of 11-dehydrocorticosterone (**A**, **B**), corticosterone (A-**D**), dexamethasone (**E**, **F**), or aldosterone (**G**, **H**) in the presence or absence of 1 μM of MR antagonist spironolactone (**C**, D, G, H) or 1 μM of GR antagonist RU-486 (C-F) for 24 h, as indicated. (A, C, G, E) Quantification of IL-6 mRNA expression by real-time RT-PCR. Data represent ratios of IL-6 mRNA to GAPDH control mRNA from treated cells normalized to the values obtained from cells incubated with vehicle (DMSO). (B, D, F, H) Quantification of IL-6 protein expression by ELISA. Data represent mean ± SD from three independent experiments. **P <0.01, ***P <0.005.

Since glucocorticoids are known as potent anti-inflammatory drugs, we next determined the concentration dependence of IL-6 expression and compared the effects of 11-dehydrocorticosterone, corticosterone, and dexamethasone. The potent GR agonist dexamethasone suppressed IL-6 mRNA and protein expression in a concentration-dependent manner (Figure 3A, B). Unlike the GR-selective ligand dexamethasone, 11-dehydrocorticosterone (upon conversion to corticosterone by 11β-HSD1) showed a bi-phasic response with peak stimulatory effects at about 50 nM and potent suppression at concentrations higher than 250 nM. Neither spironolactone nor RU-486 at a concentration of 1 μM inhibited 11β-HSD1 enzyme activity (measured as conversion of radiolabeled cortisone to cortisol in cell lysates). At 20 μM, spironolactone showed weak inhibition with 78 ± 14% remaining activity, and in the presence of RU-486 remaining activity was 69 ± 9%, thus excluding that the observed effects of the antagonists on IL-6 expression were due to 11β-HSD1 inhibition. A similar bi-phasic response, with maximal stimulation at 25 nM, was obtained using corticosterone. The stimulatory effect, but not the suppressive effect, could be prevented by co-treatment with the MR antagonist spironolactone (Figure 3C). The bi-phasic response to corticosterone of IL-6 expression and suppression by spironolactone was confirmed on the protein level using ELISA (Figure 3D). High corticosterone concentrations, that is 250 nM, decreased IL-6 protein levels. The GR antagonist RU-486 did not affect the corticosterone-induced stimulation of IL-6 mRNA and protein expression. Importantly, at 250 nM corticosterone, which suppressed IL-6 expression, co-incubation with RU-486 caused an increase in IL-6 mRNA and protein expression (Figure 3C, D). This suggests that at higher glucocorticoid concentrations GR prevents MR-mediated activation of IL-6 production and that GR blockade results in pronounced MR-mediated stimulation of production of pro-inflammatory cytokines. Dexamethasone did not affect IL-6 mRNA expression at 100 nM but resulted in a decrease at higher concentrations (Figure 3E). Interestingly, IL-6 protein production was significantly decreased at 100 nM dexamethasone (Figure 3F), suggesting an inhibition of IL-6 translation or decreased protein stability. The reason for the high concentration of dexamethasone needed to suppress IL-6 expression remains unclear; however, since intact cells were used, an efflux pump may be involved. As expected, spironolactone did not affect the dexamethasone-mediated effects (data not shown), whereas they were reversed by RU-486 (Figure 3E, F). Opposite effects were obtained upon incubation of BV-2 cells with the MR ligand aldosterone, which induced IL-6 mRNA and protein expression, whereby these effects were fully reversed by co-treatment with spironolactone (Figure 3G, H).

### Effect of corticosteroids on IL-6 expression is mediated through NF-κB

**Figure 4:**
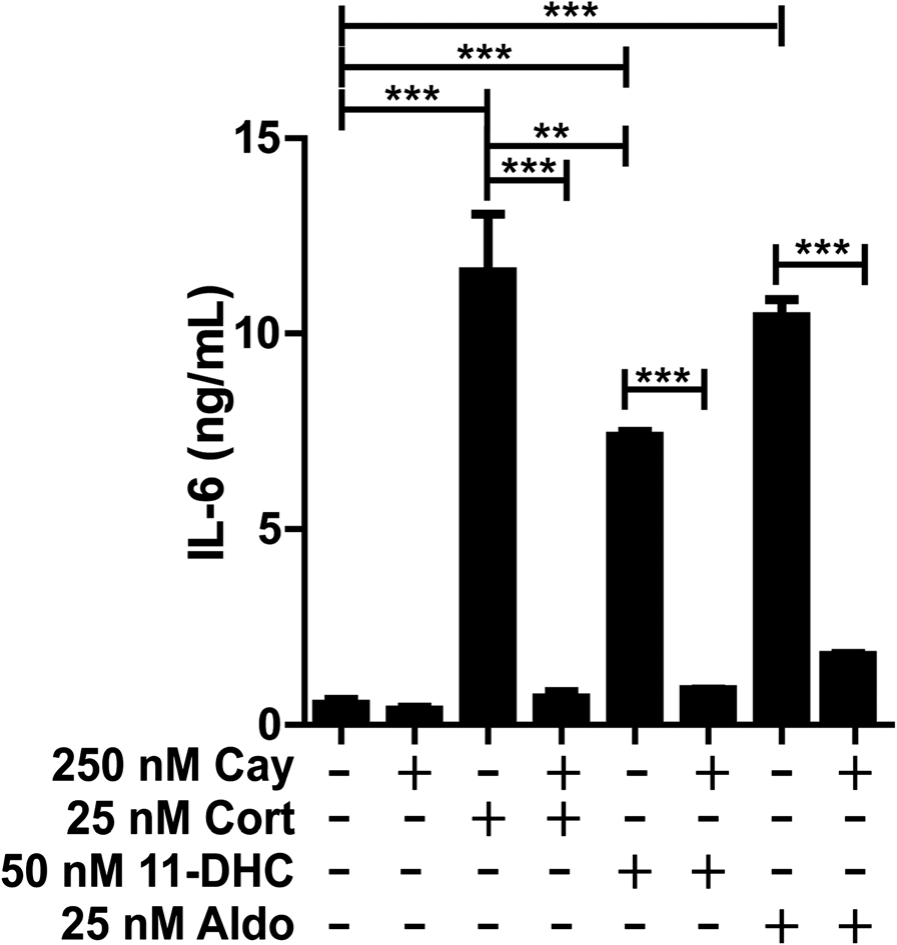
**Blockage of corticosteroid**-**dependent IL-6 induction by inhibition of NF-κB.** BV-2 cells were pretreated with 250 nM of the NF-κB inhibitor Cay-10512 for 1 h, followed by incubation with 25 nM corticosterone, 50 nM 11-dehydrocorticosterone, or aldosterone for another 24 h. IL-6 protein was measured by ELISA. Results represent mean ± SD from three independent experiments. **P <0.01 and ***P <0.005.

**Figure 5:**
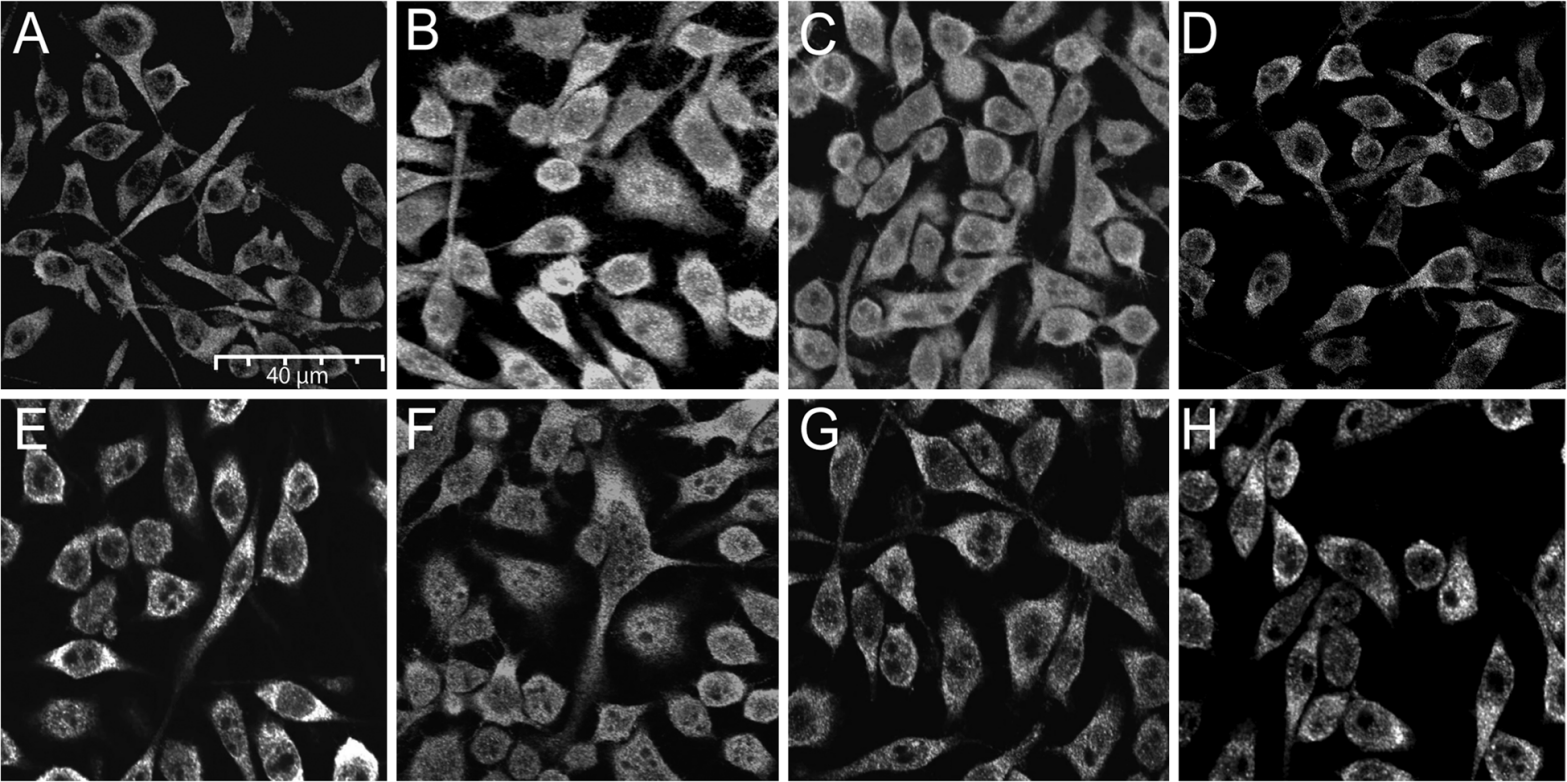
**Corticosterone**- **and aldosterone**-**mediated NF**-**κB translocation is dependent on MR.** BV-2 cells were treated for 24 h with vehicle (**A**), 50 ng/mL TNF-α (**B**), 25 nM corticosterone (**C**), 25 nM corticosterone and 1 μM spironolactone (**D**), 25 nM corticosterone and 250 nM Cay-10512 (**E**), 50 nM aldosterone (**F**), 50 nM aldosterone and 1 μM spironolactone (**G**), or 50 nM aldosterone and 250 nM Cay-10512 (**H**). The localization of the p65 subunit of NF-κB was visualized by immunofluorescence microscopy using a primary antibody against p65 and a secondary ALEXA fluor 488 labeled antibody (magnification 400×).

Next, we tested whether the stimulation of IL-6 production by low/moderate concentrations of 11-dehydrocorticosterone, corticosterone, and aldosterone is dependent on NF-κB activation. Treatment of cells with the NF-κB translocation inhibitor Cay-10512 diminished the corticosteroid-mediated IL-6 production (Figure 4), suggesting that MR-dependent activation of NF-κB is involved in the stimulation of IL-6 expression. To visualize NF-κB activation, immune-fluorescence staining using antibody against the p65 subunit of NF-κB was performed. As shown in Figure 5, incubation of cells with 25 nM corticosterone or 25 nM aldosterone enhanced the presence of p65 in the nuclei, whereby the corticosteroid-induced NF-κB translocation could be prevented by co-treatment with either spironolactone or Cay-10512.

**Figure 6:**
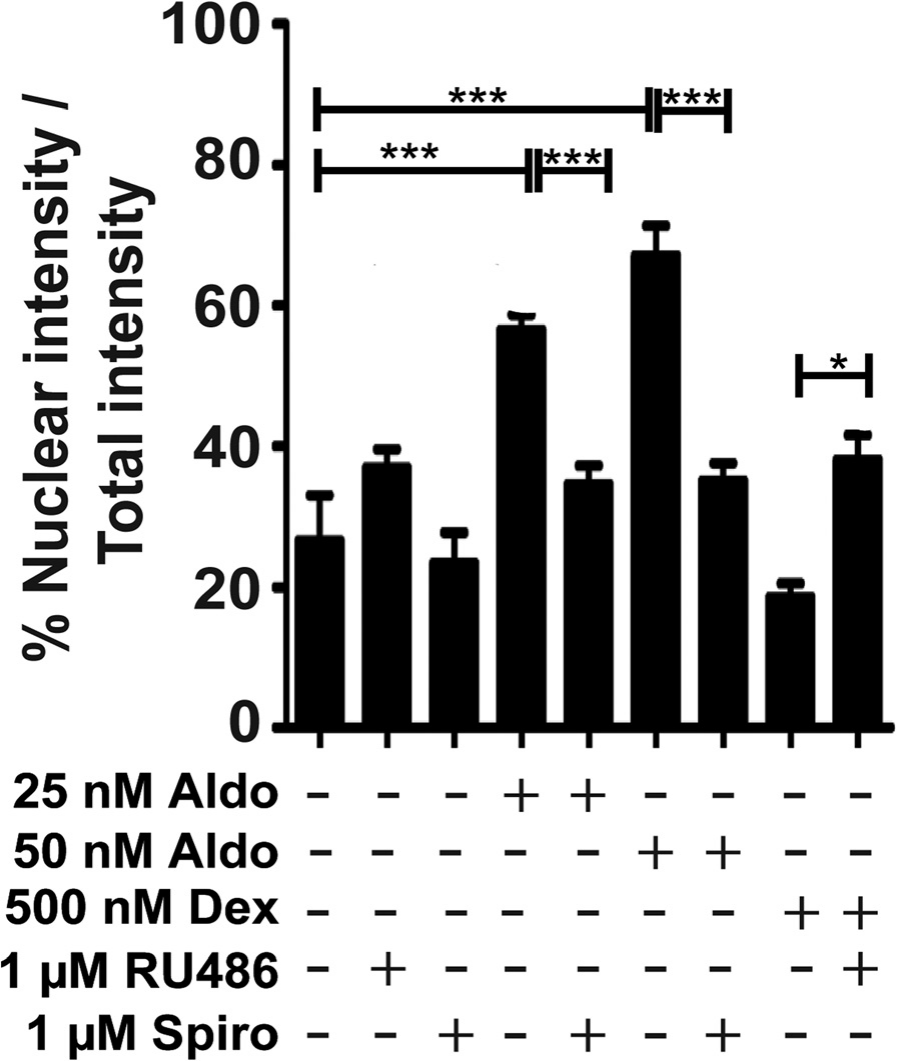
**Differential effects of MR and GR agonists on NF-κB translocation.** BV-2 cells were treated with aldosterone or dexamethasone in the presence or absence of 1 μM RU-486 or 1 μM spironolactone for 24 h, followed by analysis of the intracellular localization of p65 by Cellomics ArrayScan HCS imaging system. The ratio between the intensity of nuclear p65 fluorescence and total cellular p65 fluorescence was quantitated. Results represent mean ± SD from three independent experiments. *P <0.05 and ***P <0.005.

**Figure 7:**
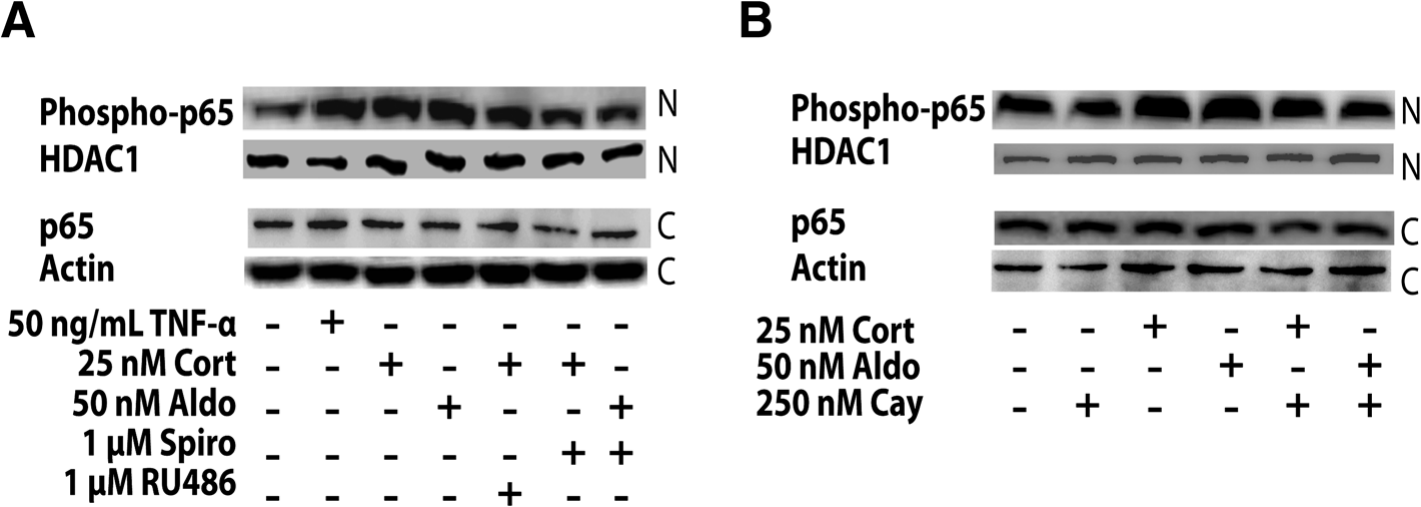
**Nuclear-cytoplasmic distribution of NF-κB p65 determined by western blotting.** BV-2 cells were treated for 24 h with 50 ng/mL TNF-α, 25 nM corticosterone, or 50 nM aldosterone in the absence or presence of 1 μM spironolactone or 1 μM RU-486 (**A**), or in the presence of 250 nM Cay-10512 (**B**), followed by preparation of nuclear and cytoplasmic fractions, separation of proteins by SDS-PAGE and analysis of p65 and phospho-p65 expression by western blotting. Actin served as a loading control. A representative blot from three independent experiments is shown.

The MR-dependent NF-κB activation was further analyzed by determination of the distribution of p65 using Cellomics ArrayScan high-content imaging (Figure 6). Incubation with aldosterone enhanced the presence of p65 in the nucleus, an effect that was blocked by spironolactone. In contrast, incubation with dexamethasone diminished the presence of p65 in the nucleus, and co-incubation with the GR antagonist RU-486 prevented this effect. The subcellular distribution of p65 was further assessed by western blot analysis of cytoplasmic and nuclear fractions of BV-2 cells. Incubation of cells in the presence of corticosterone or aldosterone led to elevated amounts of the phosphorylated form of p65 in the nucleus, an effect that was prevented by co-treatment with spironolactone (Figure 7A) or Cay-10512 (Figure 7B).

### Differential modulation of TNFR2 expression by MR and GR

**Figure 8:**
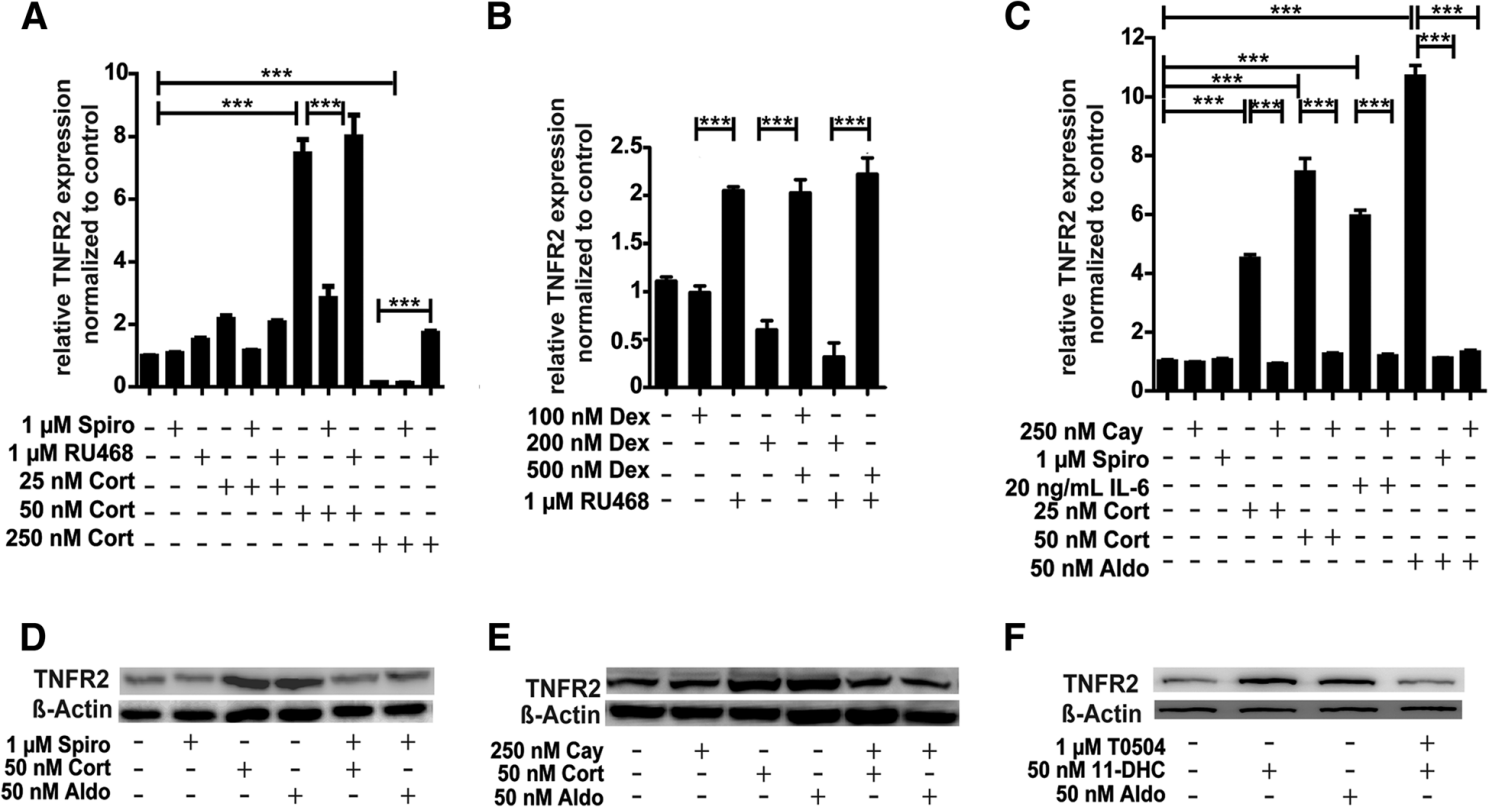
**Activation of MR but not GR enhanced TNFR2 expression by a NF**-**κB**-**dependent mechanism.** BV-2 cells were treated for 24 h with corticosterone (**A**, **C**, **D**, **E**), 11-dehydrocorticosterone (**F**), dexamethasone (**B**), or aldosterone (C-F), in the presence or absence of 1 μM spironolactone (A, C, D), 1 μM RU-486 (A, B), or 250 nM NF-κB inhibitor Cay-10512 (C, E). The impact of 11β-HSD1 was assessed by co-incubation of cells with 1 μM T0504. TNFR2 mRNA was quantitated by real-time RT-PCR. Data (mean ± SD from three independent experiments) represent ratios of TNFR2 mRNA to GAPDH control mRNA from treated cells normalized to the values obtained from cells incubated with vehicle (DMSO). For western blot analysis (D, E, F) equal protein amounts were loaded and probed for TNFR2 with actin as a loading control. A representative blot from three independent experiments is shown. ***P <0.005.

The sensitivity of microglia cells to pro-inflammatory cytokines such as TNF-α is dependent, among other factors, on the expression levels of membrane surface receptors like TNFR2 that control intracellular signaling pathways. As observed for IL-6 expression, low/moderate concentrations of corticosterone, that is 25 nM and 50 nM, induced TNFR2 mRNA expression but high concentrations of 250 nM or higher decreased its expression (Figure 8A). The induction by low/moderate corticosterone was reversed by co-treatment with the MR antagonist spironolactone, whereas GR antagonist RU-486 abolished the suppressive effect of high corticosterone. The selective GR ligand dexamethasone had an opposite effect with a concentration-dependent suppression of TNFR2 mRNA expression that was reversed by co-incubation with RU-486 (Figure 8B). As observed for low corticosterone, TNFR2 mRNA expression was induced upon treatment with aldosterone (Figure 8C). The induction of TNFR2 expression by corticosterone (50 nM) and aldosterone (25 nM) was confirmed on the protein level by western blotting (Figure 8D). Moreover, we found that the inhibitor of NF-κB translocation Cay-10512 was able to completely abolish the induction of TNFR2 mRNA expression by IL-6, low/moderate glucocorticoids or aldosterone (Figure 8C). Western blotting revealed the potentiation of TNFR2 protein expression by 50 nM corticosterone and 50 nM aldosterone, and further demonstrated the requirement of NF-κB activation for this induction (Figure 8E). 11-dehydrocorticosterone similarly induced TNFR2 expression, which was prevented by inhibition of 11β-HSD1 (Figure 8F).

### IL-6 and low glucocorticoids synergistically increased 11β-HSD1 mRNA expression and reductase activity

**Figure 9:**
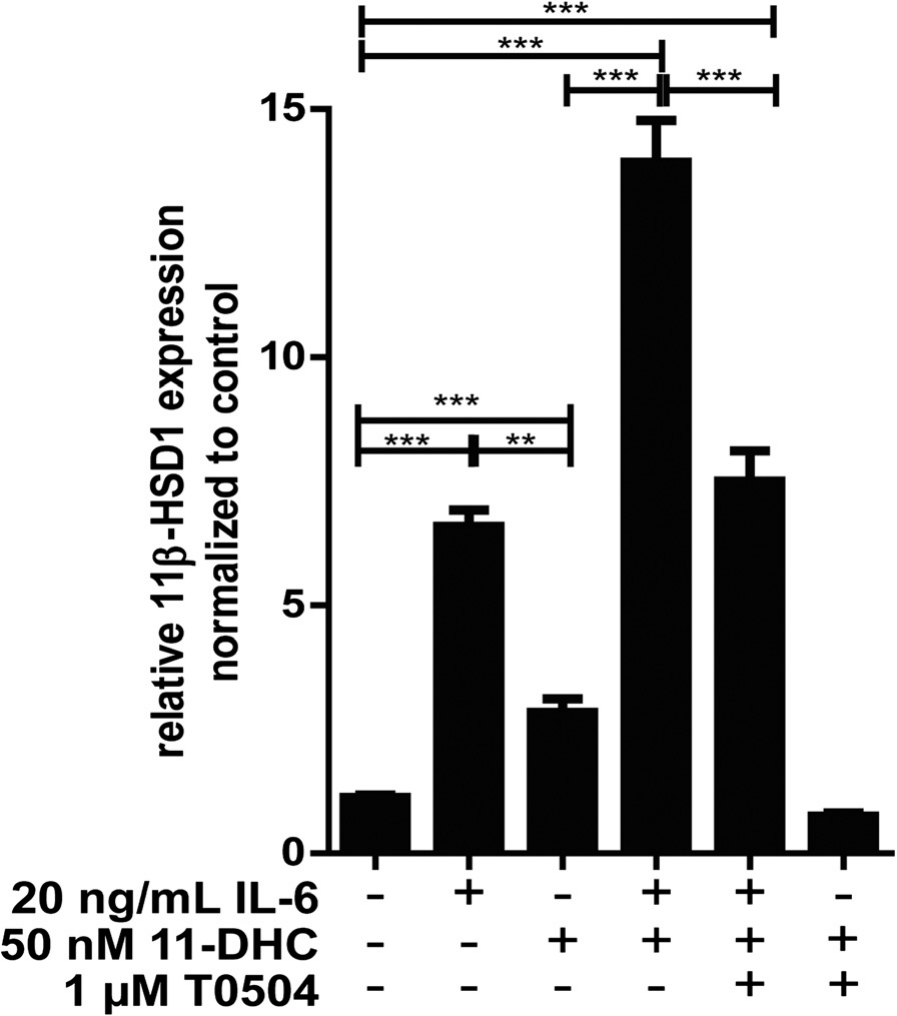
**Potentiation of the IL-6-dependent increase of 11β-HSD1 mRNA expression by low concentrations of 11-dehydrocorticosterone.** BV-cells were pretreated with 1 μM 11β-HSD1 inhibitor T0504 where indicated for 1 h, followed by addition of 50 nM 11-dehydrocorticosterone with or without 20 ng/mL IL-6 and incubation for another 24 h. 11β-HSD1 mRNA was determined by real-time RT-PCR. Data (mean ± SD from three independent experiments) represent ratios of 11β-HSD1 mRNA to GAPDH control mRNA from treated cells normalized to the values obtained from cells incubated with vehicle (DMSO). **P <0.01, ***P <0.005.

Glucocorticoids showed a bi-phasic effect on the production of pro-inflammatory cytokines, depending on activation of MR only, at low concentrations, or both MR and GR at higher concentrations. Thus, an elevation of the intracellular concentration of active glucocorticoids by 11β-HSD1 may play an important role in the resolution of inflammation by causing a shift from MR- to GR-mediated regulation of pro-inflammatory cytokine expression. As shown in Figure 9, incubation of BV-2 cells with 20 ng/mL of IL-6 resulted in increased 11β-HSD1 mRNA expression, which was strongly enhanced by co-incubation with 50 nM 11-dehydrocorticosterone. Pharmacological inhibition of 11β-HSD1 prevented the effects of 11-dehydrocorticosterone. To test whether incubation with IL-6 would enhance 11β-HSD1 enzyme activity, we measured the conversion of radiolabeled cortisone to cortisol. Due to technical reasons cortisone instead of 11-dehydrocorticosterone was used to study the effect on enzyme activity. Both IL-6 and cortisone resulted in increased 11β-HSD1 reductase activity, which was strongly enhanced by co-incubation with IL-6 and cortisone (data not shown), indicating that the changes observed on the mRNA level are translated to enzyme activity.

**Figure 10:**
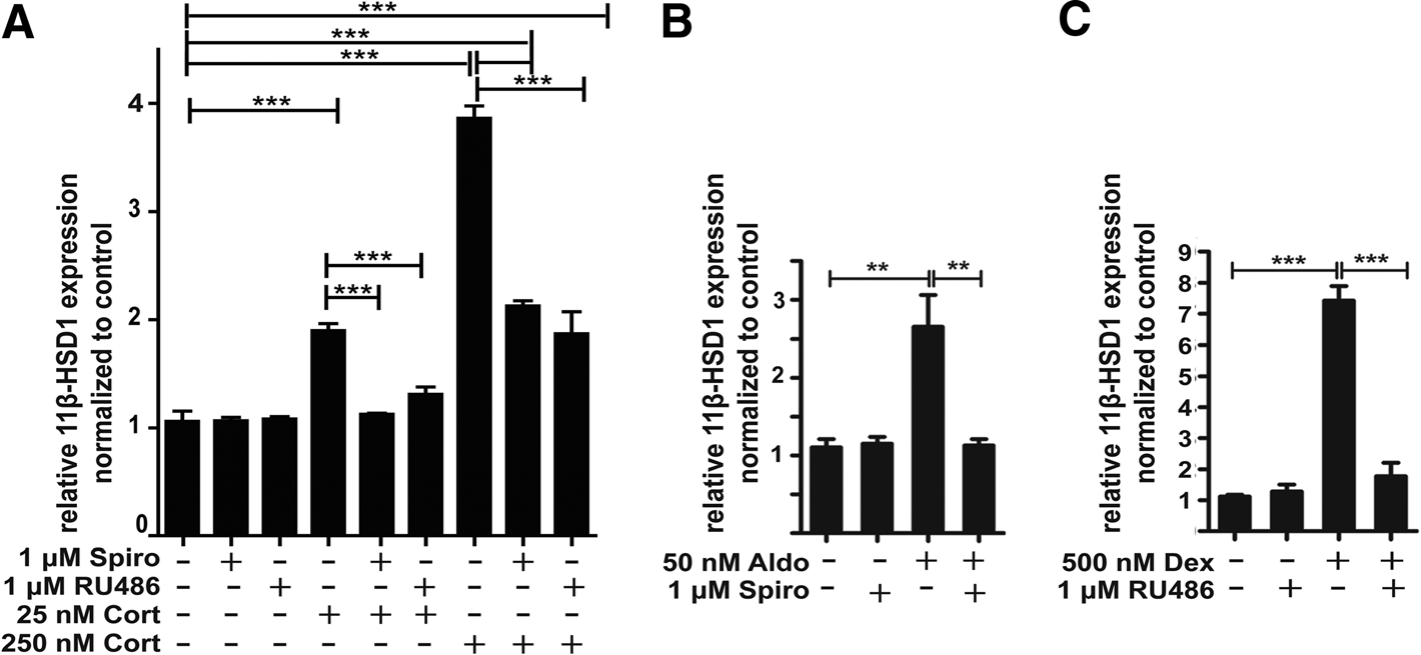
**11β-HSD1 mRNA expression is enhanced by both MR and GR activation.** BV-2 cells were treated for 24 h with corticosterone (**A**), aldosterone (**B**), or dexamethasone (**C**) in the presence or absence of 1 μM spironolactone (A, B) or 1 μM RU-486 (A, C). 11β-HSD1 mRNA was quantitated by real-time RT-PCR. Data (mean ± SD from three independent experiments) represent ratios of 11β-HSD1 mRNA to GAPDH control mRNA from treated cells normalized to the values obtained from cells incubated with vehicle (DMSO). **P <0.01, ***P <0.005.

Interestingly, while low corticosterone in the absence of IL-6 showed weak stimulatory effects on 11β-HSD1 expression, higher concentrations resulted in a more pronounced induction of expression (Figure 10). Spironolactone and RU-486 both were able to block the induction of 11β-HSD1 expression. Both, treatment of cells with aldosterone and dexamethasone enhanced 11β-HSD1 mRNA expression. Together with the fact that low and high glucocorticoid concentrations led to increased 11β-HSD1 expression, the results suggest a feed-forward regulation including both MR and GR.

## Discussion

A recent comparison of the murine BV-2 cell line with primary mouse microglia cells revealed highly overlapping gene expression profiles upon stimulation with LPS, although the response in BV-2 was generally less pronounced [25]. Approximately 90% of the genes induced by LPS in BV-2 cells were also induced in primary microglia, and 50% of the genes were also affected in hippocampal microglia following *invivo* stimulation of mice by intracerebroventricular LPS injection. These observations indicate that the BV-2 cell line is a suitable model of murine microglia to study neuroinflammatory parameters.

In the present study, we employed BV-2 cells as a macrophage/microglia cell model to characterize the impact of endogenous and synthetic corticosteroids on NF-κB activation and on IL-6 and TNFR2 expression. We found that BV-2 cells functionally express MR, GR, and 11β-HSD1, and our results emphasize the importance of a well-balanced activity of MR, which stimulates pro-inflammatory mediators, and GR that counteracts these effects. The selective MR ligand aldosterone exclusively resulted in NF-κB activation and upregulation of IL-6 and TNFR2 expression, whereas the selective GR ligand dexamethasone had opposite effects. The concentration of aldosterone to activate MR in BV-2 cells was approximately one order of magnitude higher than anticipated based on its K_d_ of about 0.5 nM. The effect on IL-6 protein levels was more pronounced than that on mRNA expression, indicating increased translation and/or protein stability. Similar observations were made using dexamethasone, for which K_d_ values for GR of 3–4 nM have been reported [5,35]. While 100 nM dexamethasone readily suppressed IL-6 protein it had no effect on mRNA expression. As a possible explanation an efflux pump expressed in BV-2 cells may lower the actual intracellular concentration of aldosterone and dexamethasone.

Corticosterone and 11-dehydrocorticosterone (upon conversion to corticosterone by 11β-HSD1) stimulated the expression of IL-6 and TNFR2 and activated NF-κB at low/moderate concentrations by acting through MR, whereas higher concentrations exerted suppressive effects by acting through GR. Over 95% of the circulating corticosterone is bound to transcortin and albumin. Under normal conditions peak corticosterone concentrations in mice and rats in the unstressed state range between 250 nM and 500 nM, thus assuming that the free fraction in plasma reaches concentrations up to 25 nM. The intracellular concentrations may differ significantly from this value depending on uptake and 11β-HSD-dependent metabolism. In the presented experiments, we used 25 nM as a low and 250 nM as a high corticosterone concentration in culture medium containing 10% FBS. Despite the reduced amount of serum proteins present in the culture medium, the capacity should be sufficient to bind most of the 25 nM corticosterone added, probably resulting in a free steroid concentration below 2 nM. Nevertheless, the MR with a K_d_ of 0.5 nM for corticosterone is expected to be occupied, whereas the GR with a K_d_ of 5 to 10 nM is probably not activated, thus reflecting the situation under normal physiological conditions. In contrast, at 250 nM the binding capacity of the FBS present in the culture medium is probably saturated, resulting in high unbound corticosterone levels, reflecting levels reached during stress conditions and leading to occupation of GR.

Upon further increasing corticosterone from concentrations needed for maximal induction of IL-6 expression, a rapid decline was observed (Figure 3A, B). This may be explained by the higher expression levels of GR compared with MR, suggesting that occupation of few GR molecules may be sufficient to suppress MR activity. It is not clear at present whether GR suppresses MR function by competing for coactivators/corepressors, by competing for binding sites on the promoter of a given target gene, or by formation of heterodimers. Interestingly, RU-486 enhanced IL-6 and TNFR2 expression in the absence of added steroids (Figure 3E, Figure 8B). In preliminary experiments using transfected cells, we observed ligand-independent MR activity that was lowered upon co-expressing GR. RU-486 might act as an inverse agonist and induce a conformational change upon binding to GR, which may prevent heterodimer formation or, alternatively, GR may compete with MR for a corepressor protein, thereby increasing MR activity.

Nevertheless, the results suggest a tightly controlled and coordinated action of MR and GR in the regulation of NF-κB activity and the production of and sensitivity to pro-inflammatory cytokines in microglia cells. Pro-inflammatory cytokines such as TNF-α, IL-1β, and IL-6, and subsequent activation of NF-κB, lead to elevated expression and activity of 11β-HSD1, which results in enhanced local levels of active glucocorticoids (this study, and [18]). The fact that both GR and MR promote 11β-HSD1 expression may represent an important feed-forward regulation of glucocorticoid activation in order to increase the intracellular concentration of active glucocorticoids and to shift the balance from an initially predominantly MR-mediated stimulation to a GR-mediated suppression of inflammation. The enhanced local glucocorticoid activation may be necessary for the resolution of inflammation. The suppression of pro-inflammatory cytokines and NF-κB activity upon activation of GR should then allow normalization of 11β-HSD1 expression and activity.

Evidence for a stimulation of inflammatory parameters by low to moderate glucocorticoid concentrations was also obtained from recent experiments with murine 3 T3-L1 adipocytes [1]. Decreased adiponectin mRNA and increased IL-6 mRNA were observed upon incubation of 3 T3-L1 adipocytes with 100 nM corticosterone or cortisol. These effects were partially reversed by treatment with the MR antagonist eplerenone but not by GR antagonist RU486, suggesting that MR activation was responsible for the observed effects. Furthermore, 100 nM of the glucocorticoids increased NADPH oxidase subunit p22 mRNA levels and decreased catalase mRNA levels, which were reversed by co-treatment with eplerenone. However, the authors did not test the effects of various concentrations, nor was the role of 11β-HSD1 addressed.

Other investigators observed elevated 11β-HSD1 mRNA and activity in 3 T3-L1 adipocytes that were treated with LPS, TNF-α, or IL-1β [2]. Pharmacological inhibition of 11β-HSD1 diminished the TNF-α-induced activation of NF-κB and MAPK signaling. Their study, however, did not assess the role of MR and the impact of higher glucocorticoid concentrations. At high glucocorticoid levels, pharmacological inhibition of 11β-HSD1 might abolish GR activation, thereby promoting MR-mediated pro-inflammatory effects. Since actual glucocorticoid concentrations in intact microglia cannot be measured, it will be important to assess the effects of 11β-HSD1 inhibitors on neuroinflammation *in vivo* in future studies. Nevertheless, these observations are in line with our findings in microglia cells that low/moderate glucocorticoid concentrations mainly act through MR, thereby promoting inflammatory parameters.

During acute inflammation, TNF-α acts through membrane-bound TNF-receptors on macrophage and microglial cells, leading to activation of transcription factors such as NF-κB and AP1, which can induce a second wave of pro-inflammatory cytokines, including TNF-α, IL-1β and IL-6. TNFR2 is highly expressed on microglia cells and plays an important role in the regulation of innate immune response following brain injury on infection [36]. An elevated expression of TNFR2 upon MR activation, as observed in the present study, is expected to result in a higher sensitivity and more pronounced response to external pro-inflammatory stimuli.

Disruption of MR- and GR-mediated regulation of gene transcription and interaction with other transcription factors can occur at several levels. Reduced GR activity and/or enhanced MR activity, thus exacerbating inflammation, may be caused by the presence of xenobiotics differentially modulating receptor activity, post-translational receptor modifications, altered function of receptor-associated proteins, or altered protein stability [30]. The pro-inflammatory cytokines TNF-α, IL-1β, and IL-6 were shown to activate the HPA axis [37], thereby enhancing circulating glucocorticoids and exerting suppressive effects through GR activation. However, high levels of TNF-α have been associated with glucocorticoid resistance [38]. Upon excessive HPA activation, a downregulation of GR activity, probably caused by altered phosphorylation of the receptor and reduced protein stability [38,39], with concomitant glucocorticoid resistance has been observed, which may cause a shift from GR- to MR-mediated glucocorticoid effects. GR blockade by administration of RU486 or elimination of glucocorticoids by adrenalectomy sensitized C57BL/6 mice to low-dose TNF-α [38]. Moreover, hepatic GR-deficient mice showed significantly higher levels of IL-6 in response to TNF-α treatment.

Glucocorticoid-resistance represents a major problem in chronic inflammation, including rheumatoid arthritis, ulcerative colitis, Crohn’s disease, atherosclerosis, cystic fibrosis, and chronic obstructive pulmonary disease [40]. An impaired suppression by GR may lead to chronically enhanced MR activity. It remains to be investigated whether MR antagonists may prove beneficial in these diseases.

Increasing evidence indicates that neuroinflammation contributes to neuronal degeneration and the progression of Parkinson’s disease [41,42]. Activated microglial cells and increased expression of pro-inflammatory mediators have been found in the substantia nigra of patients. Interestingly, elevated circulating cortisol levels were measured in Parkinson’s disease patients together with decreased GR expression in the substantia nigra [43]. Selective ablation of GR in macrophage/microglia exacerbated the loss of dopaminergic neurons induced by 1-methyl-4-phenyl-1,2,3,6-tetrahydropyridine (MPTP), enhanced the production of pro-inflammatory parameters, and diminished the expression of anti-inflammatory mediators. Based on the findings of the present study, we hypothesize that the potentiation of neuroinflammation in GR-deficient states is due to an impaired balance of pro- and anti-inflammatory mediators as a result of a dysbalance of MR and GR activity. The role of MR in Parkinson’s disease and whether MR antagonists may prove useful in the treatment of this disease remain to be investigated.

## Conclusions

**Figure 11:**
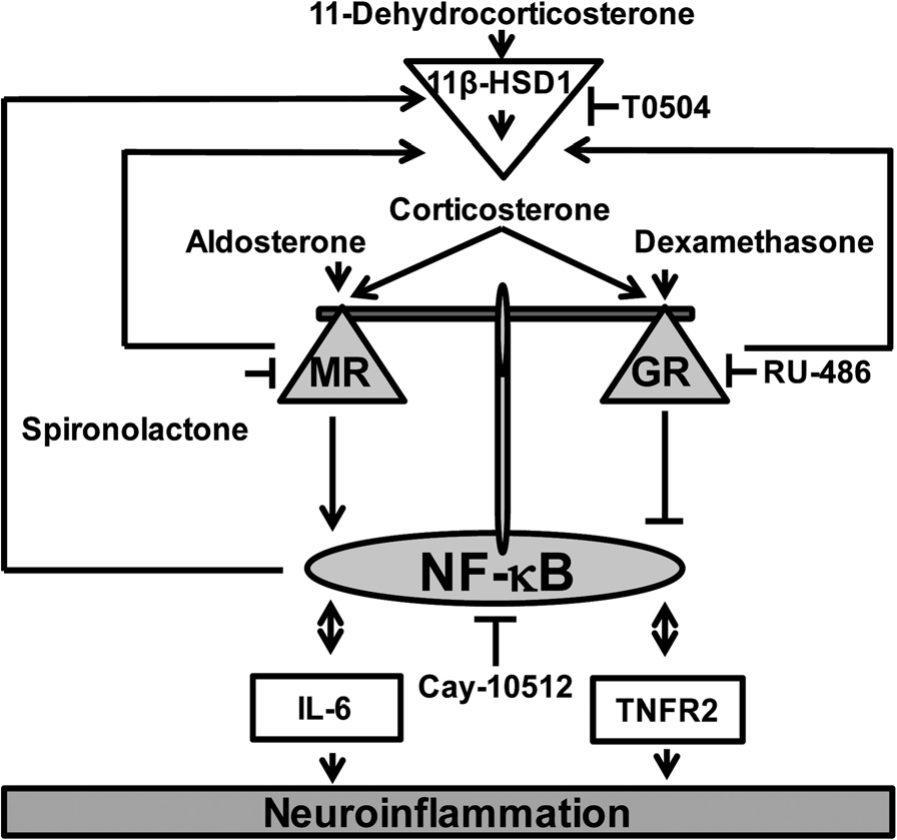
**Model for the role of MR and GR in regulating neuroinflammation in BV-2 cells.** This study suggests that MR promotes a neuroinflammatory response mediated through NF-κB activation, which can be blocked by activation GR. The control of local glucocorticoid availability by 11β-HSD1 is important in regulating the fine-tuning of the balance between MR and GR activity. Corticosterone binds with high affinity to MR, followed by binding to GR with lower affinity. 11β-HSD1 inhibitors (such as T0504) block the differential effects of 11-dehydrocorticosterone on MR and GR. IL-6 stimulated 11β-HSD1 expression, suggesting that IL-6 is involved in a regulatory feed-forward mechanism to adjust the local levels of active glucocorticoids and therefore the balance between MR and GR. IL-6 and TNFR2 activation both lead to the activation of NF-κB, and their own expression is upregulated upon NF-κB activation.

BV-2 cells represent a suitable microglia cell model for studying effects of endogenous and synthetic corticosteroids on inflammatory parameters (Figure 11). These cells co-express MR and GR, which differentially modulate NF-κB activity, the production of pro-inflammatory cytokines and membrane-surface receptors involved in the sensitivity to TNF-α. The availability of active endogenous glucocorticoids in these cells is tightly controlled by 11β-HSD1, whose expression and activity is induced by pro-inflammatory cytokines and subsequent NF-κB activation. By enhancing intracellular concentrations of active glucocorticoids, 11β-HSD1 impacts on the balanced regulation of MR- and GR-mediated responses and may play a crucial role in resolution of inflammation. Impaired function of each of the three proteins is expected to cause disturbed inflammation.

## Abbreviations

11β-HSD1: 11β-hydroxysteroid dehydrogenase 1; BCA: Bicinchoninic acid; DEPC: Diethyl pyrocarbonate; DMEM: Dulbecco’s Modified Eagle Medium; DMSO: Dimethyl sulfoxide; DTT: Dithiothreitol; ELISA: Enzyme-linked immunosorbent assay; FBS: Fetal bovine serum; GAPDH: Glyceraldehyde 3-phosphate dehydrogenase; GR: Glucocorticoid receptor; HCS: High-content screening; HDAC-1: Histone deacetylase 1; HEPES: 4-(2-hydroxyethyl)-1-piperazineethanesulfonic acid; HRP: Horseradish peroxidase; IL-6: Interleukin-6; LPS: Lipopolysacharide; MR: Mineralocorticoid receptor; NF-κB: Nuclear factor kappa-light-chain-enhancer of activated B cells; PBS: Phosphate buffered saline; PVDF: Polyvinylidene difluoride; SDS-PAGE: Sodium dodecyl sulfate polyacrylamide gel electrophoresis; TNF-α: Tumor necrosis factor-alpha; TNFR2: Tumor necrosis factor receptor 2.

## Competing interests

The authors declare that they have no competing interests.

## Authors’ contribution

BC and AO designed the study. BC performed the experiments and analyzed the data. DK, LN, and ZB helped planning experiments and analyzing the data. BC, DK, and AO wrote the manuscript. All authors read and approved the final manuscript.

## Supplementary Material

Additional file 1 **Table S1.** Real-time PCR primers. Click here for file
