# Supplementary material for: Mineralocorticoid and glucocorticoid receptors differentially regulate NF-kappaB activity and pro-inflammatory cytokine production in murine BV-2 microglial cells
Source: J Neuroinflammation. 2012 Nov 28;9:260. doi: 10.1186/1742-2094-9-260 (PMC3526453; doi:10.1186/1742-2094-9-260)
Supplement: Additional file 1 — Table S1. Real-time PCR primers. [file 1742-2094-9-260-S1.doc]

**Table 1S**

Real-time PCR primers

| **Genes** | | **Primers** | **Sequences** |
| --- | --- | --- | --- |
| GAPDH  (mouse) | | forward | CTCGTGGAGTCTACTGGTGT |
|  |  | reverse | GTCATCATACTTGGCAGGTT |
| 11β-HSD1  (mouse) | | forward | GGGATAATTGACGCCCTAGC |
|  |  | reverse | TGAGGCAGGACTGTTCTAAG |
| TNFR2  (mouse) | forward | | GTGCTGTTGCCCCTGGTTAT |
|  | reverse | | GAGTAGACTTCGGGCCTCCAC |
| IL-6  (mouse) | forward | | GGAGGCTTAATTACACATGTT |
|  | reverse | | TGATTTCAAGATGAATTGGAT |
| TNF-α  (mouse) | reverse | | TTCTGTCTACTGAACTTCGG |
|  | reverse | | GTATGAGATAGCAAATCGGC |
